# Industry strategies in the parliamentary process of adopting a sugar-sweetened beverage tax in South Africa: a systematic mapping

**DOI:** 10.1186/s12992-020-00647-3

**Published:** 2020-12-10

**Authors:** Safura Abdool Karim, Petronell Kruger, Karen Hofman

**Affiliations:** SAMRC Centre for Health Economics and Decision Science - PRICELESS SA, School of Public Health, University of Witwatersrand, Johannesburg, South Africa

**Keywords:** Corporate political activity, Sugar tax, Public policy process, Sugar-sweetened beverage tax, SSBs, South Africa

## Abstract

**Background:**

In 2016, the South African government became the first in the African region to announce the introduction of an SSB tax based on sugar content as a public health measure to reduce obesity. This tax was introduced against the backdrop of South Africa having a large sugar production and SSB manufacturing industry, as well as very high unemployment rates. The introduction of fiscal measures, such as a SSB tax, has been met with well-coordinated and funded opposition in other countries.

**Methods:**

The aim of this study is to describe and analyse the arguments and strategies utilised by industry during policymaking processes to oppose regulatory actions in LMIC. This study analyses arguments and strategies used by the beverage and related industries during the public consultation phase of the process to adopt the South African SSB tax.

**Results:**

Industry opposition to the SSB tax was comprehensive and employed several tactics. First, industry underscored its economic importance and the potential job losses and other economic harms that may arise from the tax. This argument was well-received by policymakers, and similar to industry tactics employed in other middle income countries like Mexico. Second, industry discussed self-regulation and voluntary measures as a form of policy substitution, which mirrors industry responses in the US, the Caribbean and Latin America. Third, industry misused or disputed evidence to undermine the perceived efficacy of the tax. Finally, considerations for small business and their ability to compete with multi-national corporations were a unique feature of industry response.

**Conclusions:**

Industry opposition followed both general trends, and also introduced nuanced and context-specific arguments. The industry response experienced in South Africa can be instructive for other countries contemplating the introduction of similar measures.

**Supplementary Information:**

The online version contains supplementary material available at 10.1186/s12992-020-00647-3.

## Background

The link between sugar-sweetened beverages (SSBs) and obesity and the broader impact of SSBs on health have been well-documented [1–10]. Taxes on SSBs are increasingly being recognised as an effective measure to reduce obesity and prevent non-communicable diseases (NCDs) [11–17]. In recent years, several countries and jurisdictions have adopted SSB taxes as a cost-effective measure which can be used to prevent the growing burden of NCDs [13, 18–20].

Interventions which aim to reduce consumption harmful products, such as tobacco and alcohol, are often controversial or met with opposition. SSB taxes are frequently viewed as detrimental to the economic interests of industries involved in the SSB value chain (all industries involved in the lifecycle of producing SSBs from sugar cane growing to the final distribution and sale of SBBs). Related policies are consequently met with substantial opposition from industries and corporations [21–26]. During global efforts to introduce tobacco control, this opposition was comprehensive, well-funded and coordinated across many actors [21, 25, 26]. In recent years, a similarly coordinated opposition has arisen from large food and beverage companies, particularly multinational corporations (MNCs) [23–28]. The SSB industry followed this trend [6, 21, 28–30]. This opposition can have a significant impact on the policy-making process and can often undermine public health efforts to address obesity [22, 28, 31]. The role and influence of industries is of concern given the new focus of these companies on expanding into LMIC as growth markets.

Opposition from the SSB industry has included a wide range of arguments and tactics from industry, though these have varied to some extent based on the context of the country. Opposition to the New York soda portion cap policy, for example, ranged from legal challenges to arguments that such measures curtail freedom of choice and autonomy [32]. A review of industry responses to policies aiming to reduce SSB consumption in the United States described active opposition by industry to regulatory measures as well as an emphasis on self-regulation [28]. More recently, opposition to the adoption of a similar tax in the United Kingdom argued that it would be ineffective in reducing obesity and that SSBs were an inappropriate target [33]. Industry opposition in Mexico focused on how the SSB tax would harm small businesses, negatively impact employment and disproportionately affect the poor as it was regressive, in addition to being ineffective in reducing obesity [27].

In 2016, the South African government became the first in the African region to announce the introduction of an SSB tax based on sugar content as a public health measure to reduce obesity [34]. After a lengthy process, which included substantial public consultations, the Health Promotion Levy, an excise tax of about 11% - below the recommended and initially proposed 20% - was introduced [35]. The responses from Treasury indicate that these reductions and other changes to the tax were made in response to submissions from industry actors primarily relating to potential job losses [36]. Prior to this process, it was unclear which arguments and strategies would be utilised by industry actors to influence the policymaking process within the African context. The South African context was distinct from several other countries that had adopted an SSB tax insofar as there is a longstanding, but weakening sugar production industry, in addition to and related to a large SSB manufacturing industry which would potentially be impacted [37]. It is also noteworthy that South Africa was experiencing the 9th highest unemployment ranking in the world, with a 26,5% unemployment rate during the legislative process for the SSB tax [38, 39].

For these reasons, there is value in examining the policymaking process and industry arguments to understand context-specific arguments that may be raised should countries similar to South Africa decide to implement adopt an SSB tax or related policy.

This study systematically analyses the strategies and tactics used by the beverage and related industries in the policymaking process to oppose the adoption of an SSB tax in South Africa. The aim of this study is to describe and understand the strategies utilised by the food and beverage industry to oppose regulatory actions that would reduce consumption of their products and how these arguments were tailored to a low- and middle-income setting.

## Methods

### Study design and theoretical framework

This study sought to identify and analyse the arguments of industry actors during the public participation processes of the South African SSB tax to understand its tactics and strategies. We used an adaptation of the Policy Dystopia Model by Ulucanlar et al. [40] designed to describe the corporate political activity of the tobacco industry. The adapted model, created by Mialon et al. [41], proposes a framework to identify corporate political activity in the food industry. The model proposed by Ulucanlar et al. distinguishes between discursive strategies and instrumental strategies [40]. Discursive strategies refer to argument-based strategies or the narrative of the industry. Instrumental strategies refer to actions and techniques of industry to influence policy-making. This distinction is carried through into the adapted model [41]. We used this adapted model to analyse formal public submissions made on the taxation proposal at different stages of the law-making process. Once the activities had been identified, we mapped the broad argument-based strategies, as well as the action-based strategies that were disclosed during the parliamentary submissions.

### Data collection

**Fig. 1:**
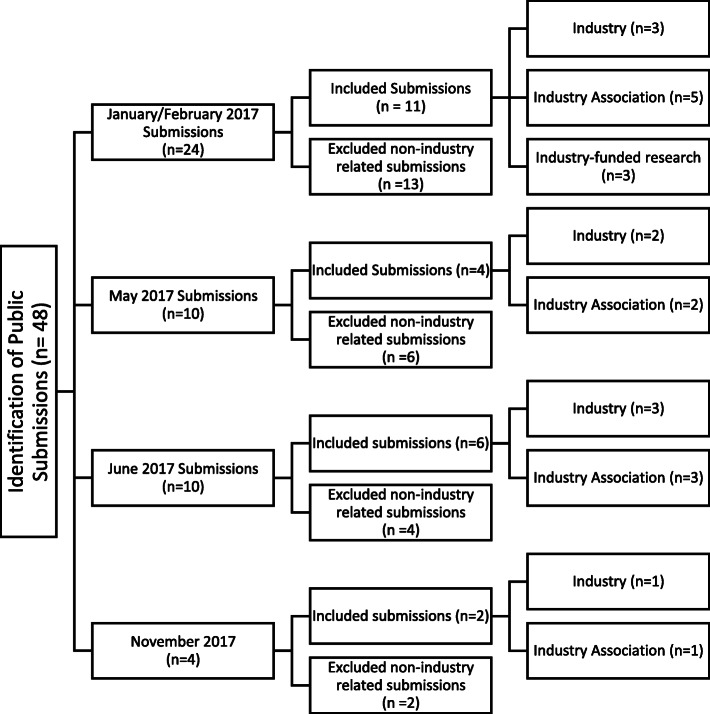
Screening process for industry-related submissions

Our data was collected from the Parliamentary Monitoring Group (PMG) database, a digital, open-access database that collates all of South Africa’s parliamentary activity. We used the bill tracker to find a timeline of events related to the SSB tax once a formal bill was developed in February 2017 and thereby identify the public participation processes related to the SSB tax [42]. There were five [5] public hearings where oral and written submissions could be made. Two of these hearings preceded the bill but was managed by parliament as it was aimed at informing the draft of the bill. We reviewed all public submissions made during these five hearings (*n* = 48). SAK and PK coded and screened submissions based on the category of stakeholder that made the submission. We included submissions made by industry, industry associations. We also included submissions we categorised as “industry funded research” which comprised of submissions from actors that were either specifically commissioned by industry to conduct research related to the SSB taxation policy or research groups who were funded by industry (*n* = 23) and excluded all others (Fig. 1). For purposes of identifying industry-funded research, we relied on the content of the submissions which did disclose whether the submission itself was commissioned and funded by industry actors or if the group making the submission received funding from industry. Supplemental Annexure 1 contains a complete list of the submissions included. SAK and PK inductively double-coded the submissions for arguments and tactics utilised by different actors.

### Data analysis

Once the submissions had been coded by arguments and themes, we utilised the adapted Policy Dystopia Model [41] to categorise the arguments according to different discursive strategies (See Table 2 below) and instrumental strategies (See Table 3 below). Given the nature of the data reviewed in this study, instrumental strategies can only be discerned through voluntary disclosure or description thereof by industry actors. The study design therefore allows for a more detailed discussion of discursive strategies. Utilising qualitative analysis, we were then able to identify common utilised strategies and techniques.

## Results

### Overview

An overview of the industry submissions made on the SSB tax is outlined in Table 1 and shows that majority of industry actors were opposed to the tax. A quarter of the actors were supportive of the tax broadly but opposed the parts of the tax that targeted their products or financial interests. For example, the South African Fruit Juice Association and Pioneer Foods, whose primary beverage product are 100% fruit juices, did not oppose the adoption of an SSB tax insofar as it would apply to SSBs like carbonated beverages, but opposed the inclusion of 100% fruit juices as taxed beverages. The submissions made by industry actors related to the fruit juice industry comprised a significant proportion of the submissions made and included industry-funded research to motivate for the exclusion of 100% fruit juices from the SSB tax. Concentrates and mixtures manufacturers were another sector that did not oppose the tax but opposed its application to their products.

**Table 1:** Overview of industry submissions stance on the SSB tax

Industry actor submissions (*n* = 23) (100%)	Opposed (*n* = 16) (69,57%)	Oppose Specific Part (*n* = 6) (26,09%)	Neutral (*n* = 1) (4,35%)	Support (*n* = 0) (0%)
Industry (*n* = 9)	**5**	**4**	**0**	**0**
Sugar industry (*n* = 1)	1			
Carbonated beverage industry (*n* = 1)	1			
Fruit juice and concentrates industry (*n* = 5)	1	4		
Other (=2)	2			
Industry association (*n* = 11)	**10**	**1**	**0**	**0**
Sugar industry (*n* = 4)	4			
Carbonated beverage industry (*n* = 3)	3			
Fruit juice and concentrates industry (*n* = 1)		1		
Other (=3)	3			
Industry-funded research (*n* = 3)	**1**	**1**	**1**	**0**
Funded by sugar industry (*n* = 0)				
Funded by carbonated beverage industry (*n* = 1)	1			
Funded by fruit juice and concentrates industry (*n* = 1)			1	
Funded by other (*n* = 1)		1		

Source: Author’s Construction

A large proportion of submissions came from industry associations representing the views of their members, while only a few industry actors opted to file independent submissions. A number of submissions came from industries related to the SSB industry, such as sugar producers and a package manufacturer, while the carbonated beverage industry and related associations made four [4] submissions.

### Analysis of discursive and instrumental strategies

Tables 2 and 3 outline the common discursive strategies and instrumental strategies and techniques adopted by industry, respectively. Discussion surrounding the economy and jobs and framing the debate on diet- and public health-related issues were prevalent discursive strategies during the parliamentary process.

**Table 2:** Discursive Industry Strategies Identified in the South African SSB Tax Adoption Process

Domain	Practice	• Specific Mechanism Observed in Public Submissions
The economy	Stress the number of jobs supported and the money generated for the economy	• Stress job creation and economic contribution
Expected food industry costs	Policy will lead to reduced sales/jobs	• Stress job creation and economic contribution
	Cost of compliance will be high	• Highlight the administrative burden associated with regulation
Frame the debate on diet- and public health-related issues	Stress the good traits of the food industry	• Emphasize existing actions combatting obesity • Reference philanthropic activities seeking to help Small and Medium Enterprises (SMEs) and vulnerable communities
	Shift the blame away from the food industry and its products, e.g. focus on individual responsibility, role of parents, physical inactivity	• Emphasising that obesity is a complex problem that requires different interventions
	Promote industry ´s preferred solutions: education, balanced diets, information, public private initiatives, self -regulation (reformulation)	• Refer to existing activities such as public-private partnerships to address obesity • Undertake voluntary actions to reduce obesity

Source: Adapted from [41]

**Table 3:** Instrumental Industry Strategies Identified in the South African SSB Tax Adoption Process

Domain	Practice	• Specific Mechanism Observed in Public Submissions
Information Management	Production	• Fund Research
	Amplification	• Cherry Pick Data
	Suppression	• Emphasize doubt in Science • Criticize evidence
	Credibility	• Pay scientists as spokespersons
Coalition Management	Establish relationships with key stakeholders	• Involvement with NEDLAC
	Seek involvement in the community	• Philanthropic activities
	Opposition fragmentation and destabilization	• Endorse submissions from other actors • Repeat messaging from other actors’ submissions
Direct involvement and influence in policy	Actor in government decision making	• Provision of technical support through commissioned research
Legal actions	Use legal action (or the threat thereof) against public policies or opponents	• Challenge the processes and procedures used to adopt the tax • Attempt to delay implementation of the tax

Source: Adapted from [41]

Regarding instrumental strategies, we observed the use of industry commissioned research as a method to manage information through production. In addition, the reliance on public-private partnership in the form of the National Economic Development and Labour Council (NEDLAC), a coalition between labour, industry and government, acted as a significant contributor to industry garnering support from different constituencies. Industry actors argued there was non-compliance with legislative procedure and processes as well as the manner in which the tax would discriminate between products in detail. The arguments related to procedure and processes were used to motivate for delaying the implementation of the tax. We did not observe opposition fragmentation and destabilization. Rather, we observed that industry actors would harmonise their arguments and messaging, often endorsing and/or referencing the submissions of another industry actor to buttress their own submissions.

Regarding gaps, we found minimal evidence of financial incentives, indirect lobbying efforts or other methods of direct involvement and influence in policy. However, the reasons for this are more likely linked to the limitations of the data analysed. A notable gap in the practices of industry related to the governance arguments emphasising autonomy and freedom of choice were rarely made by industry actors.

### Key arguments against the tax

We identified six major argument themes across industry submissions (outlined in Table 4).

**Table 4:** Key Industry Arguments Opposing the Tax

Importance of Industry	• Claims of job losses • Contribution of industry to economy • Vulnerability of sectors, specifically sugar farming
Tax will harm the poor	• How industry supports SMEs and vulnerable groups • Tax is regressive
Promotion of self-regulation and voluntary actions in lieu of SSBs taxation	• Suggest alternative government measures • Emphasis support for NCD prevention • Highlight existing voluntary industry actions
Misuse of evidence to argue the tax is inappropriate	• Commissioning favorable research • Dispute efficacy of the tax • Undermine existing evidence • Reframe existing evidence • Highlight doubt and lack of certainty
Delaying tactics	• Argue procedural non-compliance • Motivate to delay tax until more evidence is available • Claim insufficient public participation
Other negative impacts of the tax	• Administrative burden • Question motives behind the tax • Tax is discriminatory

#### Stress the importance of industry to the economy

Industry actors uniformly stressed their economic contributions to the South African economy as a main discursive strategy. Many claimed contributing to the upliftment of small and medium enterprises (SMEs), informal traders and vulnerable communities. These actors used a range of arguments and evidence to demonstrate the significance of SSBs in the South African economy. This included emphasising the amount of money generated by the industry, the number of jobs created and the amount of tax already paid to the government.

Actors highlighted the knock-on effect an SSB tax would have both on upstream and downstream actors in the value chain from the negative impact on sugar cane producers to the losses informal (spaza) shops would suffer as a result. A few of the actors, largely those involved in sugar and SSB production, also argued that the South African market was particularly vulnerable.

*“The proposed tax on sugar sweetened beverages will have substantial unintended economic consequences, at a time when the economy is facing strong headwinds curtailing our ability to enhance our contribution to broader economic growth, job creation and sustainability.”* – Beverage South Africa, an SSB industry association

A majority of the actors emphasised that there would be significant job losses arising from the tax. Some industry associations argued that the job loss projections by Treasury were flawed because they failed to account for job losses in the informal sector.

*“ [T]he proposed SSB tax will reduce the GDP by R1.85bn with unavoidable job losses. The largest loss is expected to be experienced in the informal sector where an anticipated 4000-6000 closures of informal outlets is foreseen, outlets where SSBs are estimated to contribute 17% of revenue and 30% of margin to Spaza stores. ”* – Beverage South Africa

“*[B]etween 18-24000 jobs are likely to be lost. This is in contrast to Treasury’s estimates of between 5-7000 job losses, which does not take into consideration the informal sector. In [our] view, even these conservative estimates would be devastating in the context of South Africa’s chronically high levels of unemployment, poverty and inequality. Alternate solutions therefore need to be considered.”* – Business Unity South Africa, an association representing a broad range of sectors and industry actors.

Many industries emphasised their contributions to both the informal and rural economies, arguing that an SSB tax would cause job losses in the informal sector and hurt rural economies.

*“The industry is valued at R12 billion and a large percentage of this income is invested into rural economies. Furthermore, sugarcane farmers are an integral part of the value chain of rural economies…””* – South Africa Canegrowers’ Association (SACA)

The sugar industry also raised broader challenges being faced by the industry such as the prolonged drought and cheap exports from other countries, arguing that the SSB tax would hurt an already weak and ailing industry.

The fruit juice industry emphasised the role of fruit production and agriculture within the South African economy. Smaller businesses in the SSB value chain, such as Boxmore Packaging (a PET bottle producer), Etsweletse Trading Solutions (a small sweetened juice producer) and small cane growers (as represented by the SACA) emphasised that they contributed significantly to the economy. Concerns were raised by a small business that the tax would leave them unable to compete with bigger market players.

*“With the introduction of the sugar tax we will no longer be able to compete and established and dominant players will always be bought irrespective of price adjustment as it has embedded itself in consumers DNA and they continue to build their brands as they have the marketing budgets” –* Etsweletse Trading Solutions

#### An SSB tax will harm the poor

Some industry actors also argued that the SSB tax would harm the poor. This harm would be through the scaling back of corporate social responsibility initiatives; reduced investment and the regressive nature of the tax.

“*The proposed tax on sugar sweetened beverages will negatively impact our initiatives in this area which has a special focus on black women and youths*” – Beverage South Africa

The potential negative economic impact of the SSB tax was used as justification by some industries, such as Beverage South Africa and Coca-Cola Beverages South Africa (Coca Cola SA) to curtail or otherwise adjust their support of SMEs and other outreach. Specifically, Beverage SA stated it would need to “reconsider its committed investments” in agriculture and the agro-processing value chain.

Some actors argued that poorer communities in South Africa benefited from SSBs as a cheap energy source and without access to SSBs, their ability to survive would be harmed.

*“The hungry and starving in our communities are many and they need affordable energy to be able to live, survive and grow. Sugar provides such a source of affordable energy.”* – Consumer Goods Council South Africa (CGCSA)

The CGCSA went on to argue that SSBs had health benefits, particularly for those working in mines or outdoors:

*“No mention is also made of the health benefits of sugar, such as its role in reducing thirst and assisting in rehydration of people suffering from dehydration due to high environmental temperatures or working conditions. These aspects are very important in a country and continent with an active mining industry and high temperatures when labourers are working outdoors and cannot drink enough water to combat dehydration."* – CGCSA

#### Promotion of self-regulation and voluntary actions in lieu of SSB taxation

Almost all industry and industry associations acknowledged that obesity was a significant health issue in South Africa, with several emphasising that they supported government efforts in addressing the problem. However, these statements of support were often followed by arguments that a tax would not be effective in addressing obesity or highlighting that addressing obesity required a “multidisciplinary” or “comprehensive” approach.

*“Canegrowers is extremely concerned about the increasing trend in obesity and NCDs in South Africa but is of the view that the targeting of an individual ingredient in a particular food product as the tax aims to do, is highly unlikely to resolve a complex health condition that requires a multi-disciplinary approach, including an improvement of the current government health care system” –* SACA

As can be observed from the quote above, these arguments were sometimes also interwoven with efforts to “shift blame” from diet, SSBs or sugar to other issues. For example, the South African Sugar Association (SASA) framed the action needed on obesity as needing to inform and assist consumers.

*“Multiple, evidence-based interventions to prevent and manage obesity in South Africa should be developed, planned, budgeted and implemented. A strong campaign is needed to accurately inform and enable the public in managing (sic) their weight” –* SASA

A few of the actors, such as the CGCSA and the SASA argued for action that placed greater emphasis on physical activity as a cause of obesity.

*“In 2009, research noted that a lack of exercise was a predominant factor in especially child obesity in South Africa. Should we not have targeted interventions that addresses the Issue of exercise which is a dominant concern in childhood obesity?”* – CGCSA

Actors with an interest in fruit juices were willing to shift the focus to the harms of SSBs, *other than 100% fruit juices*, as significant contributors to obesity and support the taxation of carbonated beverages which contain added sugars. This effectively leaves their product outside of the scope of the tax.

*“Pioneer Foods recognises that although the causes of obesity and being overweight are complex, dietary intake and food choices play an important role. Moreover, ingesting more calories than expended, results in weight gain and [SSBs] which contain added sugars, versus those which contain inherent intrinsic sugars (such as unsweetened 100% fruit and vegetable juice) provide calories but virtually no nutrients.”* – Pioneer Foods

Most industry actors proposed alternative action to the SSB tax, either by highlighting existing action the industry was taking to prevent obesity or arguing for self-regulation or voluntary actions industry could adopt in future. Beverage producers in particular, raised voluntary actions they had taken to reduce sugar consumption. Some of the voluntary actions being taken by actors such as Pioneer Foods, Coca Cola SA, members of Beverage South Africa and members of the CGCSA included improving labelling disclosures, voluntary advertising restrictions such as the restricting advertising to children, product reformulation. A number of industries also referred to their actions under the existing “Health Food Options” public-private partnership between NDOH and industry. In particular, Coca Cola SA, Pioneer Foods and Beverage South Africa all outlined their commitment to introducing products with less sugar to assist consumers in reducing their calorie intake.

Sugar producers who placed emphasis on risk factors other than sugar, reflected this in their voluntary activities. For example, the SASA, placed significant emphasis on the corporate social responsibility activities it undertook to promote physical activity.

*“SASA is concerned about the increase in obesity and NCDs in South Africa. The Association has a longstanding commitment to promoting healthy lifestyles and the prevention of NCDs. This has been demonstrated, for more than 30 years, by its investment in the health of society, especially in rural areas through the support of outdoor gyms, physical activity programmes, wellness events and nutrition education to health professionals and educators.*” - SASA

#### Misuse of evidence to undermine appropriateness of the tax

All the actors questioned the efficacy, impact or evidence supporting the adoption of an SSB tax using a number of information management strategies identified in the adapted model including questioning the evidence and emphasising doubt as methods of information suppression, and cherry picking evidence or amplification of favourable information to industry.

##### Questioning, criticising and cherry picking evidence

Most of the actors questioned the evidence relied on by both government officials and public health advocates. Significant portions of the evidence presented in support of the tax and showing its efficacy in other countries were dismissed as being either not relevant to the South African context or biased.

*“Much of the research reported is either inconclusive, one-sided or from international sources. Further research should follow all the protocols of correct, objective scientific research which requires logic also in its indication of cause and effect. This seems to be lacking in the arguments raised to justify the tax.”* - CGCSA

A significant number of actors included specific discussions of why evidence relating to the success of Mexico’s SSB tax could not be used to support the adoption of a tax in South Africa. However, even while arguing that local evidence was needed as international or foreign research could not be relied upon, actors emphasised jurisdictions were a tax had purportedly been less effective to support contentions that the tax would not be effective.

*“We are also concerned that much emphasis is placed on a country such as Mexico which implemented the tax claiming it as a success while ignoring for example normal seasonal sales variations. Countries such as Denmark, Iceland, Romania, Ireland and Belgium either did not implement the tax or repealed it. Notice should be taken of their reasons for doing so. .”* - CGCSA

Sugar producers took an even more aggressive approach to evidence, arguing that there was no research showing that sugar consumption was harmful to health.

*"The vast majority of mainstream science has shown no causal link between sugar consumption and obesity or any NCD, other than dental caries, in the absence of good oral hygiene"* – Tongaat Hulett Sugar South Africa, a large sugar producer (Tongaat Hulett)

In addition to taking an inconsistent approach in the applicability of some international research, some actors referred to the same studies in support of the contention that an SSB tax would not be effective. For example, both the SACA and Coca Cola SA refer to a 2014 study by McKinsey Global Institute which stated that taxing foods was among the least effective measures to prevent obesity.

*“[I]t is clearly specified that the tax is considered a measure to promote health, prevent disease and raise revenue. In fact, there is convincing scientific evidence that taxes on individual foods are ineffective in curbing rates of obesity. The McKinsey Global Institute report on obesity (2014), cites taxes on foods being the least effective measure to reduce obesity. It thus seems strange that if this is the purpose of the tax that the substantial amount of scientific evidence is being ignored"* – SACA

##### Commissioning research

Significantly, there were three industry-funded research submissions. These included a submission from the Rippe Lifestyle Institute funded by Kellogg and PepsiCo, research from Hahn & Hahn Inc. on behalf of Pioneer Foods and the Glycemic Index Foundation of South Africa (GI Foundation) also on behalf of Pioneer Foods. The GI Foundation is a government-approved endorsement organisation like Weigh Less, CANSA or Diabetes SA whose logo can be used by industry to endorse certain nutritional characteristics of food.

The research submitted by the Rippe Institute found “no adverse health effects from added sugars found at multiple levels of human consumption within the normal range in RCTs lasting up to 24 weeks.” On this basis, the Rippe Institute concluded that there were “no differences between sugar sweetened beverages and other sources of calories on any of these parameters.” The latter two submissions related to the inclusion of 100% fruit juices in the SSB tax. The research by Hahn and Hahn which was commissioned by Pioneer Foods took a neutral stance on the SSB tax, neither advocating for or against the tax. Pioneer Foods appeared to provide the research on what the scope of the tax ought to be as a form of “technical support from groups that are “fully independent, credible and acting in the public interest” on. The GI Foundation research took a strong position against the inclusion of 100% fruit juices in the SSB tax as “100% Fruit Juice has more in common with the health aspects of fresh fruit and vegetables, than the similarities it shares with SSB’s, provided the serving size is sensible.”

#### Delaying tactics

Delaying tactics are not expressly identified in the adapted model, however, it is foreseen in the original Policy Dystopia Model and most closely link to the instrumental strategy of direct involvement and influence in policy. It also overlaps with the use of threats of legal action as several of the arguments surrounding delay seemed to be couched in procedural requirements which could be used to attack the validity of the policy later.

A number of tactics and arguments were raised by industry actors in an attempt to delay the implementation of the SSB tax in South Africa. These arguments included that industry needed more time to prepare for the implementation of the tax, more evidence and research should be conducted before adopting a tax, there had not been sufficient public participation or stakeholder consultation, and there was procedural non-compliance in the process of adopting the tax.

##### Industry requires more time to prepare for the tax

Both sugar producers and beverage manufacturers argued that the implementation of the tax ought to be delayed to allow industry to adapt their products and prepare for the tax. Coca Cola SA stated that reducing the sugar content would be a “mammoth” task:

*“While Treasury has indicated that the 4 grams threshold was arrived as a result of stakeholder engagement, it presents a mammoth target for Coca Cola SA to attain in a short space of time”* – Coca Cola SA

Despite a number of actors protesting the applicability of the United Kingdom (UK) experience in implementing an SSB tax, particularly given their status as a high-income country, SASA referred to the time period UK policy makers afforded industry to prepare for the implementation of the tax and requested a similar time period be given to South African industry.

*“In the United Kingdom, two years’ notice has been given for the implementation of their SSBs tax regime whereas only a very short period of one year has been provided in South Africa. The industry therefore requests a longer period to adjust to the proposed tax”* – ASA

##### More evidence and research should be conducted before adopting a tax

Some industry actors argued that consideration of the tax should be halted until a Total Dietary Intake Study could be conducted to identify contributors to obesity in South Africa and this study ought to be used to identify measures to prevent obesity. In addition to this, a number of actors also suggested that extensive additional research be conducted. In their submissions, actors highlighted concern that the scientific basis for the tax was unclear and there was not sufficient evidence that could be applicable to the South African context. They suggested delaying the implementation of the tax until more research could be conducted.

##### Insufficient public participation or stakeholder consultation

A number of actors, across sectors, argued that there had not been sufficient public participation and consultation with key stakeholders in the process to adopt the SSB tax. In particular, actors emphasised a lack of consultation with both industry actors themselves as well as consumers and marginalised groups. In particular, actors argued for more extensive consultations with key stakeholders who would be most affected by the SSB tax i.e. the SSB and SSB value chain industries.

*"It would be negligent not to reiterate our concern that the background work by Treasury informing both the first and revised proposal on a sugary drinks tax has not received the required scrutiny by key stakeholders* … *Our credence is further that, much work still needs to be done for ordinary consumers, informal traders and the lowest income groups to be thoroughly consulted on the implications of the tax for them."* – Coca Cola SA

Notably, industry also attempted to utilise the NEDLAC coalition to give the NEDLAC members, including industry actors, a level of oversight over the Finance Committee-run public participation process which was independent of NEDLAC actors. Specifically, Tongaat Hulett, Pioneer Foods, Beverage South Africa and the CGCSA all argued for stronger involvement and increased consultation with NEDLAC.

*"We request [that]… the Treasury be required to carry out a comprehensive SEIAS, with proper engagement with stakeholders, to be reviewed by the partners in the NEDLAC process." –* Tongaat Hulett

In addition, industry actors attempted to prevent or delay the SSB taxation proposal from moving forward before the NEDLAC process was completed. It is worth noting that Treasury, the Department of Finance and the Department of Health are all excluded from the NEDLAC process and thus these attempts would have the effect of limiting the influence of key policymakers in decisions on whether to proceed with passing the tax.

*"We also support the [NEDLAC] consultation on the Health Promotion Levy through the NEDLAC forum. We can only humbly request that the opportunity be afforded for this consultation process to run its full course in seeking a solution that takes into account the health needs and economic impacts of any measure*" - CGCSA

##### Procedural non-compliance in the process of adopting the tax

Many of the actors requested the tax be removed from consideration and debate until an assessment of the socio-economic impact assessment, not typically a requirement for a taxation amendment bill, be conducted in full. This assessment was provided at a later stage in the participation process.

*“Insufficient consideration has been given to the full impact of the imposition of the tax, and the distressing unintended industrial, socio-economic and agricultural consequences. The imposition of the tax is an inappropriate financial instrument and will not achieve the desired reduction in obesity in South Africa”* – SASA

A number of actors placed significant emphasis on the process that was followed in housing the SSB tax within an existing excise tax framework rather than creating a new taxation mechanism for the tax in an attempt to challenge and potential have policymakers restart the process of adopting the SSB tax.

#### Other negative impacts of the tax

Some of the industry actors argued that the tax was discriminatory. The reasons and form of this argument varied depending on which sector the actor represented. For example, the sugar producers argued it was discriminatory to single out sugar as one ingredient while Coca Cola SA argued that the tax was discriminatory because it applied to only one category of products despite the fact that many products have high calories. Tiger Brands was a unique actor in the process as its interest in the tax was limited to how it applied to concentrates and in particular, they argued that the threshold and the application of the tax to undiluted concentrates and mixes was discriminatory and unconstitutional.

Some of the smaller businesses in the SSB value chain, such as Boxmore Packaging, Etsweletse Trading Solutions, along with the SACA, which represents small cane growers, raised concerns that the tax would have a differential impact of SMEs. Arguments included that the tax would have a disproportionate impact on small, black-owned enterprises and that, for these manufacturers, the effective rate would translate to being significantly more than 20%.

*“Sugar Tax undermines government’s program of developing black industrialists in that emerging black producers in the industry will inevitably have to close shop owing to the anti-competitive nature of the tax.”* - Etsweletse Trading Solutions

Many of the companies argued that the structure of the tax would result in a high administrative burden on revenue collection. However, Boxmore Packaging raised a specific argument that the tax may give rise to an illicit beverages market in the same way that the excise tax on cigarettes had created an illegal market.

*“Not only will tracking and checking sugar tax declarations and payments become a HUGE administrative burden for SARS, there is a real risk that it creates an ‘illegal’ beverages market in the informal sector.” –* Boxmore Packaging

## Discussion

The aim of this study was to describe and analyse the arguments and strategies utilised by industry during policymaking processes to oppose regulatory actions in LMIC. Our results demonstrate that the industry strategies utilised during the adoption of the SSB tax in South Africa reflected some strategies utilised previously in high income countries seeking to adopt the tax such as misuse of evidence and emphasis on voluntary actions. However, our results also reflect an approach tailored in an LMIC context like South Africa which leveraged in the country’s status as an emerging economy and the tensions between prioritising economic growth against health.

One of the most dominant discursive strategies made in the process related to the economic importance of industry, in particular the contribution of both the sugar and SSB industries to the economy as well as the potential job losses and other economic harms that may arise from the tax. This argument gained particular traction in South Africa where there are incredibly high unemployment rates. To emphasise the negative impact of the tax, many industry actors disputed the job loss projections presented by Treasury, and presented inflated job loss statistics with little to no evidence to support these assertions [43]. Industry actors also leveraged on the impact the tax would have on the informal sector and SMEs. The impact on informal traders was used to argue that the job losses associated with the tax would be substantially higher than government anticipated. SMEs emphasised the harms the tax would cause as disproportionately affecting small or informal businesses and leaving them unable to compete with large MNCs. These arguments were also canvassed outside of the formal parliamentary process in the media, often with inflated numbers [43–45]. Policy makers were sensitive to these claims about potential job losses and the final rate and structure of the tax was amended to try and alleviate the stress on industry in an attempt to mitigate potential employment consequences. The emphasis on this argument parallels the core of industry opposition in Mexico, another middle income setting [27].

Industry also framed themselves as a part of the solution to obesity with their submissions highlighting their support for action to prevent obesity and emphasising existing partnerships with the Department of Health to improve South African diets. During the policymaking process, SSB manufacturers began pre-emptively reducing the sugar content of beverages, reducing package sizes, limiting marketing to children and changing product labels [46–48]. Similar reactive actions occurred in response to proposed US regulations [2]. The emphasis on self-regulation and voluntary measures as a form of policy substitution mirrored industry responses to broader regulation of SSBs in the US as well as proposals of weaker alternatives to regulation in the Caribbean and Latin America [33, 49]. The submissions reviewed referred equally to both voluntary actions, including global commitments such as the Coca Cola pledge on marketing to children, and public-private partnerships between the Department of Health and industry actors. The expansiveness of these public-private partnerships, their entrenchment in the Department of Health’s policymaking processes and their influence were highlighted have been identified as a major source of corporate political activity in South Africa [50]. Within the SSB policymaking process, the existence of public-private partnerships to curb obesity and NCDs was used to question the need for regulation. This approach of industry utilising public-private partnerships to undermine regulations has previously occurred in Fiji [51]. This highlights the need for governments to be circumspect about engagement with industry through the creation of public-private partnerships as these partnerships may, in the future, be used to hinder the adoption of or argue for the dilution of policies targeting the corporate determinants of health.

Misuse and misrepresentation of evidence was also key industry strategy. Industry actors disputed evidence showing the efficacy and appropriateness of an SSB tax in reducing obesity as being “one-sided” or from other countries. The South African government was able to rely on locally-generated evidence and referenced this within its policy documents [36]. Similarly, supporters of the tax, such as civil society and academia, were able to rely on and present research supporting the adoption of an SSB tax which provided a strong basis to advocate for the adoption of an SSB tax [15]. Industry actors also commissioned research and disputed the applicability of research and evidence from other countries. Of particular note was the fact that some MNCs, such as Kelloggs and PepsiCo, commissioned seemingly independent research to influence the policymaking process. This is reflective of some similar tactics used the tobacco industry or the ultra-processed food industry more broadly [26, 47, 52, 53]. Fooks et al. identified substantial misuse of evidence in a sub-set of submissions made on the initial taxation policy which included disregarding extensive evidence of the efficacy of the tax and cherry picking data, all to create doubt about the efficacy of an SSB tax [54]. However, we found that actors went even further, arguing the tax would harm the poor which included arguing the tax was regressive while framing SSBs as important to poor and marginalised communities. Sugar producers advanced unsupported arguments about the health benefits of SSBs as an energy source for labourers and stressed the importance of SSBs to the poor. However, other research has shown that high acceptability of SSBs among vulnerable communities stems from industry practices such as extensive advertising and wide availability rather an innate cultural acceptability [55].

Interestingly, the governance arguments of autonomy and freedom of choice so prevalent in the context of the US and Australia [53, 56] were not raised by industry actors in South Africa. There was an express recognition of government’s role in obesity prevention and their responsibility to take action which is reflective of the political landscape in South Africa, and many other LMIC, which prioritises poverty alleviation over neoliberal ideologies [57]. However, industry messaging attempted to frame causes of obesity as an issue of personal responsibility or as caused by a lack of physical activity. Some actors also questioned government’s motives in adopting the tax as being for revenue generation mechanism rather than for health purposes, a concern echoed by poorer communities in their views and perceptions of the SSB tax [55].

Also distinct from other countries’ experiences was the lack of express reference or resort to litigation. Where litigation or the threat thereof has been a central part of industry responses to SSB regulation in New York, other US states and Australia [32, 33, 55], in South Africa this issue was limited to raising technical challenges in the policymaking process and did not result in litigation. This may be a result of previous court decisions where legislation regulating tobacco was upheld as constitutional and other decisions concerning pharmaceutical companies were decided in favour of the government [58, 59]. Consequently, countries with strong traditions of upholding the right to health or measures protecting public health may not be subject to an industry response through litigation.

The literature on corporate political activity and industry responses to the adoption of policies regulating the corporate determinants of health in South African and LMICs more broadly is still developing. However, our findings reflect previous findings from Fooks et al. [54] and Mialon et al. [50] that self-regulation, public private partnerships and misuse of evidence are key industry tactics. More significantly, our results demonstrate points of departure where industry argument in high-income settings such as infringement of autonomy may give way to concerns about employment and economic harms that are key issues in LMIC.

### Limitations

We limited our data collection to publicly available submissions. We are aware that there was an additional public submission process but these records were not publicly available and an internet search yielded only four results which did not represent a substantial proportion of comments made. In addition, reviewing the submissions and the number of repeat submissions made by industry actors, showed saturation in our sample. Our study is a limited but detailed analysis of the strategies used during only the parliamentary process. Due to the data set used, our ability to analyse the instrumental strategies was limited and dependant on voluntary disclosures. Further research could look at broader industry strategies used in other arenas such as the media and direct lobbying efforts outside the formal law-making process.

### Recommendations

Our study highlights the need for LMIC governments to articulate clear policy priorities and ensure policy coherence between health and economic development as these tensions can be leveraged in opposing an SSB tax. In addition, we recommend LMIC governments limit the role of voluntary actions and public-private partnerships with industry as these partnerships are used by industry to argue against regulatory action and binding policies. Despite the substantial body of evidence demonstrating the efficacy of SSB taxes in reducing sugar consumption and preventing obesity, industry actors continue to engage in an array of strategies to question the need for and efficacy of an SSB tax. For this reason, it is important that policy makers articulate a clear, evidence-based rationale for an SSB taxation policy. In LMIC, it may be of particular value to have local and context-specific evidence as industry actors may question the transferability or appropriateness of evidence generated in high income countries.

## Conclusion

In many respects, the response of industry actors to the adoption of SSB taxation is reflective of industry responses to SSB regulation globally. However, our study demonstrates the ways in which a response may be tailored to a specific-context. In particular contextual factors such as high unemployment and a historically prominent sugar production industry played a significant role in shaping the industry response in South Africa. As the first African country to adopt an SSB tax for public health purposes, the industry response experienced in South Africa can be instructive for other countries contemplating the introduction of similar measures.

## Supplementary Information

**Additional file 1:** **Supplemental Annexure 1:** Breakdown of Industry-related Parliamentary Submissions

## Data Availability

Not applicable.

## Abbreviations

SSB: Sugar-sweetened beverage
NCD: Non-communicable disease
MNC: Multi-national Corporation
LMIC: Low- and middle-income countries
NEDLAC: National Economic Development and Labour Council
SME: Small and medium enterprise
CGCSA: Consumer Goods Council South Africa
SACA: South Africa Canegrowers Association
SASA: South African Sugar Association
