# Supplementary material for: Industry strategies in the parliamentary process of adopting a sugar-sweetened beverage tax in South Africa: a systematic mapping
Source: Global Health. 2020 Dec 10;16:116. doi: 10.1186/s12992-020-00647-3 (PMC7725882; doi:10.1186/s12992-020-00647-3)
Supplement: Supplementary file 1 — Additional file 1: Supplemental Annexure 1: Breakdown of Industry-related Parliamentary Submissions [file 12992_2020_647_MOESM1_ESM.docx]

**Supplemental Annexure 1: Breakdown of Submissions**

| January / February 2017 | |
| --- | --- |
| Industry | - Tiger Brands - Pioneer Foods - Boxmore Packaging |
| Industry Associations | - Beverage SA - SASA - SA Fruit Juice Association - CGCSA - SACA |
| Industry Funded Research | - Rippe Lifestyle Institute - Hahn & Hahn - Glycemic Index Foundation |
| May 2017 | |
| Industry | - Etsweletse Trading Solutions - Tiger Brands |
| Industry Associations | - Business Unity SA - SASA |
| June 2017 | |
| Industry | - Coca-Cola SA - Pioneer Foods - Tongaat Hulett |
| Industry Associations | - Beverage SA - SACA - CGCSA |
| November 2017 | |
| Industry | - Tiger Brands |
| Industry Associations | - Beverage SA |
